# Binding of Acetate in the S_2_ State of the Oxygen-Evolving Complex in Photosystem II

**DOI:** 10.3390/plants15152291

**Published:** 2026-07-26

**Authors:** Julianne S. Lampert, Gourab Banerjee, Ipsita Ghosh, Jinchan Liu, Krystle M. Reiss, Richard J. Debus, Victor S. Batista, Gary W. Brudvig

**Affiliations:** 1 Department of Chemistry, Yale University, New Haven, CT 06520, USA; julie.lampert@yale.edu (J.S.L.); victor.batista@yale.edu (V.S.B.); 2 Department of Molecular Biophysics and Biochemistry, Yale University, New Haven, CT 06520, USA; 3 Department of Biochemistry, University of California, Riverside, CA 92521, USA; debusrj@ucr.edu

**Keywords:** photosystem II, oxygen-evolving complex, water oxidation, acetate binding, electron paramagnetic resonance

## Abstract

Photosynthetic water oxidation is catalyzed by the Mn_4_CaO_5_ oxygen-evolving complex (OEC) of photosystem II (PSII), where hydrogen-bonding and ion-binding networks regulate proton transfer, substrate-water delivery, and S-state advancement. Acetate binding inhibits oxygen evolution, competes with chloride, and stabilizes the S=5/2 spin isomer of the S_2_ state, but its donor-side binding site remains unresolved. Here, we combine EPR spectroscopy, pH-dependent oxygen-evolution measurements, mutagenesis, and QM/MM calculations to support a donor-side acetate-binding model and determine how acetate perturbs the OEC environment. Acetate increases the ratio of the g=4.1 to g=2 S_2_-state EPR signals in spinach PSII membranes and cyanobacterial PSII core complexes, with stronger stabilization persisting to higher pH in spinach PSII. The D1-N87A *Synechocystis* PSII variant exhibits spinach-like acetate sensitivity and pH-dependent oxygen-evolution behavior, with an effective acidic $pK_{a}$ of approximately 5.3, versus 4.2 for wild-type cyanobacterial PSII, implicating long-range perturbations of the narrow-channel hydrogen-bonding network. QM/MM calculations support acetate binding near the D1-D61/W1 region, where the acetate-bound S=5/2 isomer is only 1.0 kcal mol^−1^ higher in free energy than the S=1/2 isomer, consistent with the observed spin-isomer equilibrium shift. These results reveal how acetate perturbs proton-transfer and chloride-binding processes in PSII.

## 1. Introduction

Photosynthetic water oxidation in cyanobacteria, algae, and green plants leads to oxygen evolution, which is essential for sustaining aerobic life. This process occurs in the transmembrane protein–pigment complex photosystem II (PSII), located within the thylakoid membrane of photosynthetic organisms [1,2]. Embedded within the core of PSII is the Mn_4_CaO_5_ oxygen-evolving complex (OEC), which is the site of water oxidation catalysis [3,4]. Beyond its central role in biology, understanding photosynthetic water oxidation is important for the development of clean-energy technologies, as the OEC provides a natural model for coupling light-driven charge separation to water oxidation and the generation of energy-rich products.

**Figure 1:**
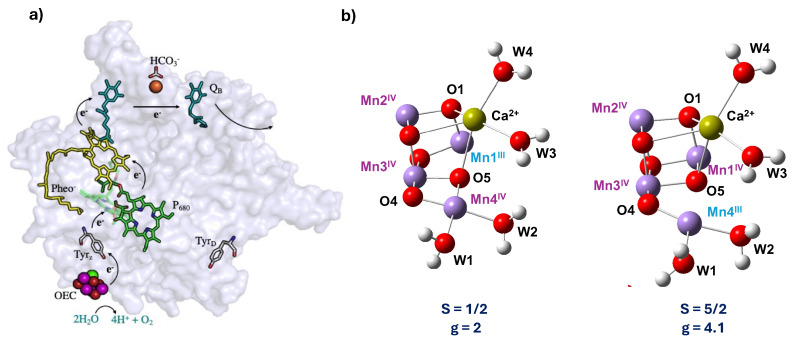
(**a**) Donor and acceptor sides of photosystem II, including the Mn_4_CaO_5_ OEC, Y_z_, P680, pheophytin, $Q_{A}$, and $Q_{B}$. Mn ions are shown in purple, O in red, Ca in green, Fe in orange, and selected residues are colored for clarity. Structure shown from 3WU2 [3]. (**b**) Representative OEC isomers associated with the g=2 and g=4.1 S_2_ state EPR signals.

Water oxidation is initiated by photoinduced charge separation at P680, a chlorophyll *a* pigment cluster that functions as the primary electron donor in PSII. Excited electrons are transferred to the acceptor side of PSII via pheophytin and the quinone electron acceptors $Q_{A}$ and $Q_{B}$ [2,4]. Reduced $Q_{B}$ forms plastoquinol, which exchanges with an oxidized quinone from the quinone pool to complete the catalytic cycle. On the donor side, electrons are replenished through water oxidation. A redox-active tyrosine, Y_z_ (D1-Y161), is oxidized by P680^+^, generating the tyrosyl radical ${Y_{z}}^{\cdot }$. This radical drives sequential one-electron oxidations of the OEC, resulting in progression through the five S-states (S_0_–S_4_) of the so-called “Kok cycle” and ultimately leading to oxygen evolution [5]. The donor and acceptor sides of PSII, including the OEC, Y_z_, P680, pheophytin, $Q_{A}$, and $Q_{B}$, are shown in Figure 1a.

Progression through the Kok cycle requires coordinated electron transfer, proton transfer, substrate-water delivery, and rearrangement of the OEC hydrogen-bonding network [6,7,8]. The OEC is embedded within an extended hydrogen-bonding network composed of water molecules, amino acid residues, and inorganic ions such as Cl^−^. This network connects the catalytic cluster to the lumen through three structured water-filled channels, often referred to as the narrow (O4) channel, the broad (Cl1) channel, and the large (O1) channel. The narrow and broad channels begin near Mn4, whereas the large channel is located closer to the Ca^2+^ ion. The narrow and broad channels contain residues and ions that are critical for proton transfer and regulation of the OEC ligand environment, and both channels have been proposed as sites of substrate-water delivery [9,10,11,12,13,14,15].

EPR spectroscopy has proven especially valuable for characterizing the electronic structure of Kok-cycle intermediates. The S_2_ state is paramagnetic and displays a characteristic multiline g=2 signal corresponding to the S=1/2 state. Several broad, featureless EPR signals have also been observed in the range of g=4.1 to 4.7, indicating that high-spin structural isomers of the S_2_ state exist in equilibrium with the low-spin conformer [16,17,18,19]. The S=1/2 isomer corresponds to a state in which the oxidation states of Mn1 to Mn4 are III, IV, IV, and IV, whereas the proposed structure for the S=5/2 spin isomer has oxidation states IV, IV, IV, III. Representative structures of these two isomers are shown in Figure 1b. Because the relative intensities of the g=2 and g=4.1 signals report on the relative stability of these two electronic configurations, the S_2_ state provides a sensitive handle for determining how changes in the protein environment perturb the OEC. Species-dependent differences in S_2_ state behavior, including differences between spinach and cyanobacterial PSII, further suggest that the surrounding hydrogen-bonding networks can tune the electronic structure of the OEC.

Small-molecule inhibitors have been employed as one approach for probing the mechanistic roles of specific regions within PSII [20,21,22]. Acetate binds to both the acceptor and donor sides of PSII, producing distinct inhibitory effects. On the acceptor side, acetate binding is chloride-independent, with an inhibition constant of $K_{I}=130\pm 40$ mM, and has been attributed to inhibition of electron transfer from ${Q_{A}}^{-}$ to $Q_{B}$, likely through competition with bicarbonate [23]. Donor-side acetate inhibition is chloride-dependent and occurs with $K_{I}=16\pm 5$ mM [23]. Donor-side acetate binding has been associated with stabilization of the S=5/2 spin isomer of the S_2_ state, inhibition of the S_2_ to-S_3_ transition, and formation of the characteristic S_2_${Y_{z}}^{\cdot }$ EPR “split signal”. The split signal arises from magnetic coupling between the S=1/2 S_2_ state and ${Y_{z}}^{\cdot }$, and its decay at 200 K produces the g=4.1 signal associated with the S=5/2 isomer [23,24,25,26]. Previous experimental and computational studies have identified a broad putative acetate-binding region on the donor side of PSII; however, the exact binding site remains unresolved, limiting mechanistic interpretation of the observed acetate-induced effects [27,28].

The higher-plant system examined in this study consists of PSII-enriched membranes prepared from spinach (*Spinacia oleracea*), whereas the cyanobacterial system consists of purified PSII core complexes from wild-type and D1-N87A *Synechocystis* PCC 6803. The D1-N87A variant provides a useful strategy for examining species-dependent differences in water oxidation chemistry and small-molecule binding [29,30]. In most cyanobacteria, D1-87 is an asparagine located in the narrow channel, whereas higher plants typically contain alanine at this position [31]. Substitution of D1-Asn87 with alanine alters chloride binding and perturbs the surrounding hydrogen-bonding network, providing a way to test whether species-dependent acetate effects arise from long-range changes in the channel network. More broadly, mutagenesis and chloride-depletion studies demonstrate that the hydrogen-bonding and ion-binding networks surrounding the OEC regulate proton transfer and water oxidation. D1-D61 and D2-K317 are particularly relevant to the present study because they participate in proton-transfer pathways associated with Mn4 and the Cl1 site [32,33,34,35,36,37].

In this study, we combine experimental and computational approaches to determine the donor-side binding site of acetate in the OEC and to understand how acetate inhibition perturbs water oxidation chemistry. We investigate the effects of acetate and pH on the g=4.1 EPR signal associated with the S=5/2 spin isomer of the S_2_ state in spinach PSII membranes and wild-type and D1-N87A *Synechocystis* PSII core complexes. We then use pH-dependent oxygen-evolution measurements to determine whether the D1-N87A mutation influences proton-transfer behavior. Finally, computational QM/MM modeling is used to evaluate the proposed acetate-binding site and provide molecular insight into the observed stabilization of the S=5/2 spin isomer and competitive binding with Cl^−^. Together, these results clarify how acetate-induced perturbations report on the hydrogen-bonding and ion-binding networks that regulate photosynthetic water oxidation.

## 2. Materials and Methods

### 2.1. Protein Isolation and Purification

The His-tagged wild-type and D1-N87A *Synechocystis* PCC 6803 strains were constructed as described previously [38]. Single colonies were selected for their ability to grow on solid media containing 5 μg/mL kanamycin monosulfate and 20 μg/mL gentamycin sulfate [38]. The cells were propagated mixotrophically in three 10 L carboys containing 5 mM glucose under warm-white fluorescent illumination at 80 μmol photons m^−2^ s^−1^ and were bubbled with 5% CO_2_ in air, as described previously [39]. The PSII extraction and purification were done under dim light conditions at 4 °C using a Ni-NTA super flow affinity resin (Qiagen, Valencia, CA, USA) as described previously [40]. The purified PSII core complexes were concentrated to ∼1 mg of Chl/mL and stored in a buffer solution containing 1.2 M betaine, 10% (*v*/*v*) glycerol, 50 mM MES-NaOH (pH 6.0), 20 mM CaCl_2_, 5 mM MgCl_2_, 50 mM histidine, 1 mM EDTA, and 0.03% (*w*/*v*) n-dodecyl *β*-D-maltoside at −80 °C. Spinach (*Spinacia oleracea*) PSII membranes were prepared as described previously [41,42] and suspended to final chlorophyll concentrations of 5.0–7.0 mg/mL in 20 mM MES-NaOH (pH 6.0), 15 mM NaCl and 30% (*w*/*v*) ethylene glycol.

### 2.2. Steady-State Oxygen Assay

Oxygen evolution was monitored with a Clark-type electrode, and the oxygen-assay chamber was maintained at 25 °C using a temperature-controlled water bath. Samples were illuminated with an Oriel 1000 W tungsten–halogen lamp. A concentration of 250 μM PPBQ with 1 mM K_3_FeCN_6_ was used as the electron acceptor and 5 μg of Chl was used in each assay.

To study the effect of pH on the oxygen-evolution activity, D1-N87A PSII core complexes were equilibrated by stirring in a buffer containing 20 mM PIPBS, 20 mM MES, 1 M sucrose, 50 mM CaCl_2_ and 10 mM NaCl with the pH ranging from 3 to 8, and then illuminated. The pH dependence of the steady-state activities was then fit to the equation:

(1) $$y=\frac{A}{\left(1+10^{pK_{a1}-pH}+10^{pH-pK_{b1}}\right)}$$

where *A* is a constant, $pK_{a1}$ is the effective acidic $pK_{a1}$, and $pK_{b1}$ is the effective basic $pK_{b1}$. For each sample type, the reported N=3 values represent technical replicate measurements performed on the same sample preparation.

### 2.3. EPR Measurements

To study the effect of acetate on the structure of the OEC, we prepared EPR samples of spinach PSII membranes and *Synechocystis* PSII core complexes in 0.5 M acetate, 50 mM MES-NaOH (pH 6.5), 10% (*v*/*v*) glycerol, 1.2 M betaine, 20 mM CaCl_2_, 5 mM MgCl_2_, and 1 mM EDTA. The samples were then concentrated to 1 mg of Chl/mL using Amicon centrifugal cells having a 100 kDa cutoff. The S_2_ state was generated by illuminating the sample with a Xe lamp for 4 min in a 200 K acetone/dry-ice bath, following a previously described procedure [43]. We performed the measurements on a Bruker ELEXSYS E500 spectrometer (Billerica, MA, USA) using an SHQ resonator and an Oxford ESR-900 continuous flow cryostat (Oxford, UK) at 7.5 K. The following EPR parameters were used for recording the spectra: microwave frequency, 9.38 GHz; modulation frequency, 100 kHz; modulation amplitude, 19.95 G; microwave power, 5 mW; sweep time, 84 s; conversion time, 41 ms; time constant, 82 ms. Each spectrum was the average of two scans. EPR spectral subtractions used to generate the $S_{2}$-minus-$S_{1}$ difference spectra were performed using OriginPro version 9.1.

### 2.4. QM/MM Modeling

QM/MM models were built from the PSII crystal structure reported by Umena et al. (PDB ID: 3WU2), following previously established protocols [3,44,45]. The system comprised approximately 2500 atoms, including all residues with Cα atoms located within 15 Å of the OEC. N-terminal acetyl and C-terminal N-methylamide capping groups were applied at the system boundary and held fixed during geometry optimization. The ∼140-atom quantum mechanical region included the OEC and its direct water ligands (W1–W4), the acetate molecule, and a selected set of 12 relevant second-shell waters and the side chains of 11 amino acid residues (D1-D61, D1-D170, D1-D342, D1-E189, D1-E333, D1-A344, CP43-E354, D1-H332, D1-H337, CP43-R357, D2-K317).

All QM/MM optimizations were performed using the ONIOM method as implemented in Gaussian16 [46,47] (Wallingford, CT, USA). The QM layer was treated using density functional theory (DFT) with the B3LYP exchange–correlation functional [48,49,50,51], employing LanL2DZ effective core potentials for Mn and Ca atoms [52,53]. The 6-31G(d) basis set was used for O and Cl, and 6-31G was used for H, C, and N atoms [54,55,56]. The relative spin configuration of the four Mn atoms was defined with broken-symmetry DFT [57,58]. The MM layer was modeled using the AMBER force field [59]. Structure visualization and analysis were performed using GaussView 6.

The QM/MM energy was obtained using the following equation: $E^{QM/MM}=E^{MM,X+Y}-E^{MM,X}+E^{QM,X}$, where *X* denotes the QM layer and X+Y denotes the entire ∼15 Å region included in the model. All states were optimized to local minima. After geometry optimization, a frequency calculation was performed to obtain a thermal correction to the Gibbs free energy, including entropic contributions, such that $G^{QM/MM}=E^{QM/MM}+G^{corr}$. A summary of all models considered is available in Table S1.

## 3. Results and Discussion

### 3.1. Acetate Binding Stabilizes the S=5/2 S_2_ State Spin Isomer in a Species- and pH-Dependent Manner

**Figure 2:**
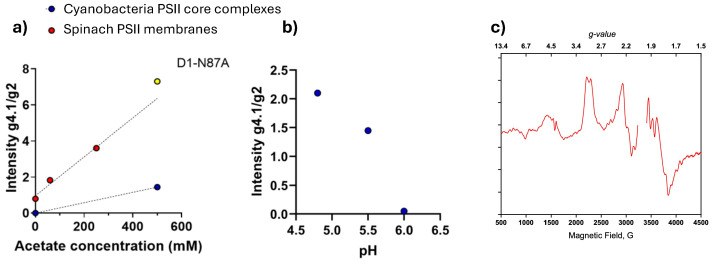
(**a**) Dependence of the ratio of the g=4.1 and g=2 S_2_ state EPR signal intensities ($R_{I}$) on acetate concentration in spinach PSII membranes (red), cyanobacterial PSII core complexes (blue), and D1-N87A PSII samples (yellow) at pH 5.5. (**b**) pH dependence of $R_{I}$ in acetate-treated cyanobacterial PSII core complexes in the presence of 500 mM acetate. Representative spectra are shown in Figure S1. Samples were suspended in 50 mM MES-NaOH buffer, 10% (*v*/*v*) glycerol, 1.2 M betaine, 20 mM CaCl_2_, 5 mM MgCl_2_, and 1 mM EDTA. (**c**) $S_{2}$ minus $S_{1}$ EPR difference spectra of acetate-treated D1-N87A PSII core complexes in the presence of 500 mM acetate at pH 5.5.

We analyzed EPR spectra of acetate-treated PSII in cyanobacterial PSII core complexes at pH 5.5. Representative EPR spectra of the acetate-treated S_2_ state are shown in Figure S2. Data for acetate-treated spinach PSII membranes and untreated cyanobacterial PSII core complexes were obtained from previous studies [23,60]. Figure 2a shows the ratio of the intensities of the g=4.1 and g=2 signals, defined as $R_{I}$, as a function of acetate concentration.

In both cyanobacterial and spinach PSII, $R_{I}$ increases with increasing acetate concentration, indicating stabilization of the S=5/2 spin isomer upon acetate binding. However, the extent of stabilization differs between the two systems and depends strongly on pH. Figure 2b shows the pH dependence of acetate binding in cyanobacterial PSII. In cyanobacterial PSII core complexes, $R_{I}$ decreases with increasing pH, and the g=4.1 signal falls below detection at pH 6.0 (Figure S1). In contrast, the g=4.1 signal persists in acetate-treated spinach PSII membranes up to pH 6.5 [23]. Thus, acetate binding shifts the S_2_ state spin-isomer equilibrium in both species, but the degree of the shift and pH range over which the S=5/2 isomer is stabilized is species dependent.

### 3.2. The D1-87 Residue Modulates Acetate Binding in PSII

Because D1-87 has been identified as the only major residue that differs near the OEC between cyanobacteria and spinach PSII (Asn versus Ala, respectively), we analyzed acetate-treated D1-N87A cyanobacterial PSII core complexes in the S_2_ state to further investigate the origin of this species difference. Figure 2c shows the corresponding EPR spectrum at pH 5.5, where both the g=2 and g=4.1 signals are observed. The resulting $R_{I}$ value is shown in Figure 2a. Unsubtracted S_1_ and S_2_ spectra are shown in Figure S3. The acetate dependence observed in D1-N87A PSII closely resembles that of spinach PSII membranes under comparable conditions. These results indicate that the species-dependent acetate-binding behavior is strongly influenced by residue D1-87 and suggest that acetate binds at a site that is structurally coupled to the hydrogen-bonding network surrounding this residue and the OEC.

The D1-N87A substitution has been shown to generate a hydrophobic cavity that likely alters the narrow-channel hydrogen-bonding network [31,61]. Interestingly, the D1-N87A variant of *Synechocystis* PCC 6803 displays S-state cycling efficiency comparable to wild-type cyanobacterial and spinach PSII, but exhibits altered chloride-binding properties that more closely resemble spinach PSII. Because the D1-87 residue is separated from the Cl site by several intervening hydrogen-bonding interactions, these observations indicate that perturbations introduced at D1-87 can propagate through the narrow-channel network and modulate the properties of distal sites. The sensitivity of acetate binding to the D1-N87A substitution therefore suggests that the acetate-binding site is not necessarily located near D1-87 itself, but is functionally coupled to a similar extended hydrogen-bonding network.

Previous pulsed EPR studies provide additional clues regarding the location of the acetate-binding site. These experiments showed that acetate binds within approximately 3.5 Å of the coupled S_2_-${Y_{z}}^{\cdot }$ state [27]. In the original study, changes in the EPR split signal were interpreted in terms of a distance of approximately 3.1 Å between the phenoxyl oxygen of ${Y_{z}}^{\cdot }$ and the non-exchangeable methyl protons of acetate. However, because the OEC and ${Y_{z}}^{\cdot }$ are magnetically coupled in this state, the split signal cannot be uniquely assigned to interactions with either center. The EPR data therefore constrain the acetate-binding site to lie within approximately 3.5 Å of either the OEC or ${Y_{z}}^{\cdot }$.

These constraints substantially narrow the range of plausible donor-side binding sites. Because acetate is chemically analogous to a protein carboxylate and shifts the equilibrium between the two S_2_ state spin isomers without dramatically perturbing the electronic structure of the Mn cluster, it is reasonable to expect that acetate binds by replacing a nearby Asp, Glu or Ala side chain. Seven residues satisfy this criterion: D1-D61, D1-D170, D1-E189, D1-D342, D1-E333, D1-A344, and CP43-E354. Among these residues, all except D1-D61 serve as direct ligands to the OEC. Acetate binding at any of these positions would therefore require disruption of a Mn–carboxylate coordination bond and substantial rearrangement of the first coordination sphere of the cluster, which is difficult to reconcile with the relatively modest spectroscopic changes induced by acetate treatment. In contrast, D1-D61 is not directly coordinated to the OEC and occupies a strategic position within the hydrogen-bonding network connecting D1-87, the Cl1 site, and the catalytic center. Taken together, these considerations identify D1-D61 as the most plausible donor-side acetate-binding site.

Related precedents are observed in other small-molecule binding studies. Both ammonia and methanol bind in the region near the narrow channel. Studies of ammonia-inhibited D1-D61A PSII samples demonstrate that ammonia interacts closely with D1-D61 [62]. Like acetate, methanol binding shifts the isomeric equilibrium of the S_2_ state, with a more pronounced effect in higher-plant PSII and in D1-N87A PSII than in wild-type cyanobacterial PSII [30,63]. These differences have been attributed to the role of D1-N87 in regulating the size, flexibility, and selectivity of the narrow channel, which has been proposed to function as an important delivery pathway for small molecules while restricting access of larger molecules to the OEC [29,30,62,64,65]. Thus, the D1-N87A mutation may enhance acetate binding by making the cyanobacterial narrow channel more plant-like, thereby increasing small-molecule access to the OEC.

### 3.3. The D1-N87A Mutation Shifts the Acidic $pK_{a}$ of PSII Toward Spinach-like Behavior

**Figure 3:**
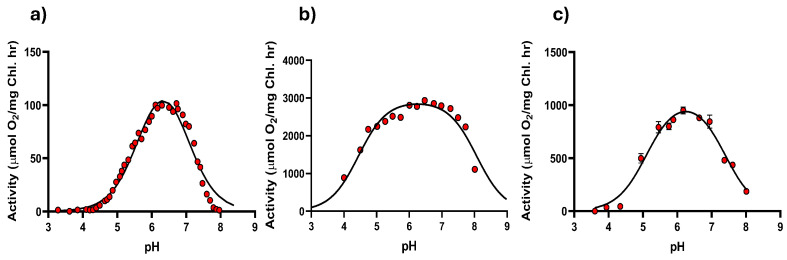
pH dependence of oxygen-evolution activity in (**a**) spinach PSII membranes, (**b**) wild-type cyanobacterial PSII core complexes, and (**c**) D1-N87A *Synechocystis* PSII core complexes. Data represent the averages of N=3 replicate measurements performed on the same sample preparation for each sample type, with standard errors. Curves were fitted using Equation (1) (Section 2) to obtain the effective acidic $pK_{a1}$ and effective basic $pK_{b1}$ values. The acidic $pK_{a}$ values are approximately 5.3 for spinach PSII and D1-N87A PSII and approximately 4.2 for wild-type cyanobacterial PSII.

Previous studies have shown that D1-D61 is involved in proton transfer, either as part of a direct proton-transfer pathway or by indirectly influencing proton-transfer energetics through modulation of the Mn4 ligand environment [33,34,36,39,44,66]. To further test whether acetate binding is coupled to the D1-D61/W1 region and whether the D1-N87A mutation influences this region through long-range interactions, we measured the pH dependence of oxygen-evolution activity in D1-N87A PSII and compared it with wild-type cyanobacterial and spinach PSII samples, as shown in Figure 3.

D1-N87A PSII exhibits pH-dependent oxygen-evolution behavior that closely resembles spinach PSII. Fitting the activity profiles to a single bell-shaped curve yields an acidic $pK_{a}$ of approximately 5.3 for both spinach PSII and D1-N87A PSII, compared to approximately 4.2 for wild-type cyanobacterial PSII. Together with the acetate-dependent EPR data, these results support a model in which the D1-N87A mutation induces spinach-like changes in the narrow-channel hydrogen-bonding network that are transmitted to the D1-D61/W1 region. This long-range coupling may alter acetate sensitivity and shift the apparent $pK_{a}$ associated with oxygen evolution. These findings suggest that interactions among D1-87, D1-D61, and the W1 hydrogen-bonding network tune proton-egress processes in a species-dependent manner. Although full modeling of this coupling is beyond the scope of the present study, these results identify this network as an important target for future investigation.

### 3.4. QM/MM Calculations Support Acetate Binding near D1-D61 and W1

**Figure 4:**
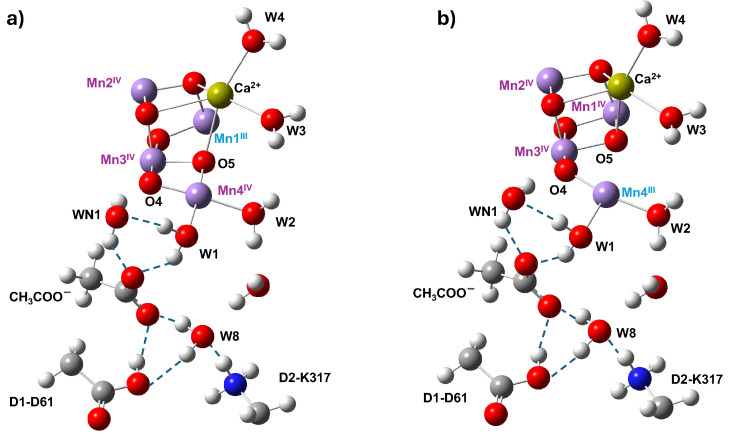
(**a**) QM/MM-optimized structure of acetate-bound PSII in the $S_{2}$ state for the g=2 isomer. For clarity, only the OEC and selected residues in the QM layer are shown. The oxidation states of Mn1–Mn4 are III, IV, IV, IV, and the spin states are αβαβ. Carbon atoms are shown in grey, Mn ions in purple, oxygen atoms in red, nitrogen atoms in blue, hydrogen atoms in white, and Ca in yellow. Selected hydrogen-bonding interactions are indicated with dashed lines. (**b**) QM/MM-optimized structure of acetate-bound PSII in the $S_{2}$ state for the g=4.1 isomer. The oxidation states of Mn1–Mn4 are IV, IV, IV, III, and the spin states are αααβ. A summary of relevant bond distances can be found in Table S2.

Figure 4a shows the QM/MM-optimized structure of the proposed acetate-bound S_2_ state. Optimization of the model resulted in proton transfer from CH_3_COOH to D1-D61 and formation of negatively charged CH_3_COO^−^, which accepts four hydrogen bonds from W1, WN1, W8, and protonated D1-D61. A state in which D1-D61 is deprotonated could not be optimized and was found to be unstable in a single-point calculation, indicating that protonation is required to stabilize this local arrangement (Figure S4). This behavior is consistent with the increased basicity expected upon loss of the strong hydrogen bond between D1-D61 and W1. These results suggest that acetate may initially bind as neutral CH_3_COOH and subsequently transfer its proton to D1-D61 as the hydrogen-bonding network reorganizes. The resulting CH_3_COO^−^ species is stabilized by hydrogen bonding to protonated D1-D61, which may help explain the pH dependence shown in Figure 2b. At higher pH, deprotonation of D1-D61 would destabilize bound acetate, leading to loss of the g=4.1 signal.

The acetate-bound S=5/2 isomer was also optimized and is shown in Figure 4b. Consistent with the experimentally observed increase in $R_{I}$ upon acetate addition, the acetate-bound S=5/2 isomer is nearly isoenergetic with the acetate-bound S=1/2 isomer. In previous non-acetate-bound QM/MM models, the S=1/2 isomer is favored by 3.1 kcal mol^−1^ while the acetate-bound S=1/2 isomer is favored by only 1.0 kcal mol^−1^. Previous models also show that the two non-acetate-bound isomers differ in the protonation state of the Mn4-bound W1 ligand [44]. For the S=1/2 isomer, Mn4^IV^ favors W1 = OH^−^ to balance the higher-valent metal center, whereas Mn4^III^ in the S=5/2 isomer favors W1 = H_2_O. Acetate binding appears to stabilize the hydrogen-bonding arrangement compatible with W1 as H_2_O, thereby shifting the equilibrium toward the Mn4^III^/S=5/2 configuration. A related but opposite perturbation has been observed for glycerol binding in the narrow channel which shifts the equilibrium toward the S=1/2 isomer. Hydrogen-bond rearrangements result in donation of an additional hydrogen bond to D1-D61, decreasing its proton affinity and favoring the W1 = OH^−^ state [22].

This interpretation is supported by optimization of an alternative S=1/2 structure in which the W1 proton resides on acetate rather than W1, shown in Figure S5. This protonation state is disfavored by 3.6 kcal mol^−1^ relative to the structure with W1 as H_2_O, as summarized in Table 1. Thus, acetate binding disfavors the protonation arrangement associated with the S=1/2 isomer and instead stabilizes a configuration more compatible with the S=5/2 state. This also suggests a mechanistic explanation for inhibition of the S_2_-to-S_3_ transition. Because D1-D61 and the Mn4-bound W1 ligand are coupled to proton-transfer chemistry during S-state advancement, acetate binding near W1 may disrupt key proton-transfer pathways or shift the $pK_{a}$ values of Mn4-bound waters [33,34,36,39,44,66]. The observation that the bound acetate has lower proton affinity than D1-D61 is likely a result of differences in the local hydrogen-bonding environment. Specifically, the strong hydrogen bond between the bound acetate and protonated D1-D61 may be responsible for the decrease in proton affinity.

Finally, the orientation of D2-K317 was examined to provide insight into the competitive relationship between acetate and Cl^−^. In the acetate-bound model, an alternative configuration was optimized in which D2-K317 reorients to form a hydrogen bond with D1-D61, as shown in Figure S6. This configuration is 4.9 kcal mol^−1^ higher in free energy than the low-spin reference model (Table 1). However, this relative free energy should be interpreted with caution because D2-K317 lies near the boundary of the QM region. Because D2-K317 also interacts with Cl^−^ in the broad channel, this rearrangement is expected to weaken chloride binding. Similar rearrangements have been observed in chloride-depleted PSII, where D2-K317 reorients toward D1-D61 to stabilize the positive charge on lysine in the absence of bound chloride [35,36]. The D2-K317–Cl^−^ interaction has also been proposed to play an important role in the deprotonation process during the S_2_-to-S_3_ transition. Therefore, acetate-induced reorientation of D2-K317 provides another possible mechanism for both acetate inhibition and the previously reported competitive relationship between acetate and chloride binding in PSII. By perturbing chloride-associated proton-transfer pathways, acetate binding may help trap the S_2_${Y_{z}}^{\cdot }$ intermediate and prevent normal advancement to the S_3_ state.

## 4. Conclusions

Together, these results support a model in which acetate binds near the D1-D61/W1 region of the OEC and perturbs the hydrogen-bonding network that controls S-state advancement. Acetate binding stabilizes the S=5/2 spin isomer of the S_2_ state in a species- and pH-dependent manner, with stronger stabilization observed in spinach PSII than in wild-type cyanobacterial PSII. D1-N87A PSII displays spinach-like acetate sensitivity and pH-dependent oxygen-evolution behavior, indicating that the residue at D1-87 modulates long-range structural rearrangements near the OEC. The effective acidic $pK_{a}$ of approximately 5.3 for D1-N87A PSII matches that of spinach PSII and is shifted relative to the value of approximately 4.2 observed for wild-type cyanobacterial PSII, further supporting long-range coupling between D1-87 and the proton-transfer network surrounding the OEC. QM/MM calculations support acetate binding near D1-D61 and W1 and provide a molecular basis for stabilization of the S=5/2 isomer. The acetate-bound S=5/2 structure is only 1.0 kcal mol^−1^ higher in free energy than the S=1/2 structure, consistent with the experimentally observed shift in the spin-isomer equilibrium. This proposed binding mode also offers a mechanism for inhibition of the S_2_-to-S_3_ transition by disrupting proton-transfer pathways and for competitive binding between acetate and Cl^−^. In particular, acetate-induced rearrangement of D2-K317 may couple perturbation of the D1-D61/W1 region to weakened chloride binding and impaired proton transfer during S-state advancement. Overall, these findings show that acetate inhibition provides a sensitive probe of the proton-transfer and ligand-binding environment of the OEC, revealing species- and mutation-dependent differences that are directly relevant to the mechanism of photosynthetic water oxidation. This long-range coupling presents an important avenue for future work aimed at understanding how interactions among D1-87, D1-D61, and the W1 hydrogen-bonding network tune proton egress and acetate sensitivity in a species-dependent manner.

## Figures and Tables

**Table 1:** Summary of selected acetate-bound $S_{2}$-state QM/MM models for which relative free energies could be directly compared. Relative free energies are reported with respect to the optimized acetate-bound low-spin reference model.

Model	Spin State	W1	Acetate	ΔG (kcal mol^−1^)
Low-spin acetate-bound $S_{2}$	S=1/2	H_2_O	CH_3_COO^−^	0.0
High-spin acetate-bound $S_{2}$	S=5/2	H_2_O	CH_3_COO^−^	1.0
Alternative W1 protonation	S=1/2	OH^−^	CH_3_COOH	3.6
D2-K317-reoriented model	S=1/2	H_2_O	CH_3_COO^−^	4.9

## Data Availability

Coordinates for QM/MM-optimized structures are available at https://github.com/JulieLampert9/Acetate (accessed on 22 July 2026).

## Supplementary Materials

The following supporting information can be downloaded at: https://www.mdpi.com/article/10.3390/plants15152291/s1, Figure S1: Acetate-treated $S_{2}$-minus-S_1_ EPR spectra of PSII core complexes at different pH values; Figure S2: Comparison of acetate-treated PSII core complex and membrane $S_{2}$-minus-S_1_ EPR spectra; Figure S3: Acetate-treated D1-N87A PSII core complex EPR spectrum; Figure S4: Spin-density redistribution upon D1-D61 deprotonation in the acetate-bound $S_{2}$ state PSII model; Figure S5: Alternative W1 protonation state in the acetate-bound $S_{2}$ state PSII model; Figure S6: Alternative D2-K317 orientation in the acetate-bound PSII model; Table S1: Summary of acetate-bound $S_{2}$-state QM/MM models; Table S2: Relevant bond distances for the hydrogen-bonding network of acetate-bound QM/MM models.

## Abbreviations

The following abbreviations are used in this manuscript:

ACE	N-terminal acetyl capping group
Chl	Chlorophyll
DFT	Density functional theory
EDTA	Ethylenediaminetetraacetic acid
EPR	Electron paramagnetic resonance
FTIR	Fourier-transform infrared spectroscopy
MES	2-(N-morpholino)ethanesulfonic acid
Ni-NTA	Nickel–nitrilotriacetic acid
NMA	C-terminal N-methylamide capping group
OEC	Oxygen-evolving complex
P680	Primary electron donor chlorophyll *a* pigment cluster in PSII
PCC	Pasteur Culture Collection
PDB	Protein Data Bank
PIPBS	Piperazine-N,N′-bis(2-ethanesulfonic acid)
PPBQ	2-phenyl-1,4-benzoquinone
PSII	Photosystem II
QM/MM	Quantum mechanics/molecular mechanics
$Q_{A}$	Primary quinone electron acceptor of PSII
$Q_{B}$	Secondary quinone electron acceptor of PSII
WT	Wild type
Y_z_	Redox-active tyrosine residue D1-Tyr161 of PSII
