# Supplementary material for: Binding of Acetate in the S2 State of the Oxygen-Evolving Complex in Photosystem II
Source: Plants (Basel). 2026 Jul 26;15(15):2291. doi: 10.3390/plants15152291 (PMC13468348; doi:10.3390/plants15152291)
Supplement: Supplementary file 1 [file plants-15-02291-s001.zip › plants-4445175-supplementary.pdf]

Supporting Information for

Binding of Acetate in the S_2 State of the Oxygen-Evolving Complex in Photosystem II

Julianne S. Lampert,[†] Gourab Banerjee,[†] Ipsita Ghosh,[†] Jinchan Liu,[‡] Krystle M. Reiss,[†] Richard J. Debus,[¶] Victor S. Batista,[†] and Gary W. Brudvig[*,‡,†]

†*Department of Chemistry, Yale University, New Haven, CT, 06520, USA*

‡*Department of Molecular Biophysics and Biochemistry, Yale University, New Haven, CT, 06520, USA*

¶*Department of Biochemistry, University of California, Riverside, California 92521, USA*

E-mail: gary.brudvig@yale.edu

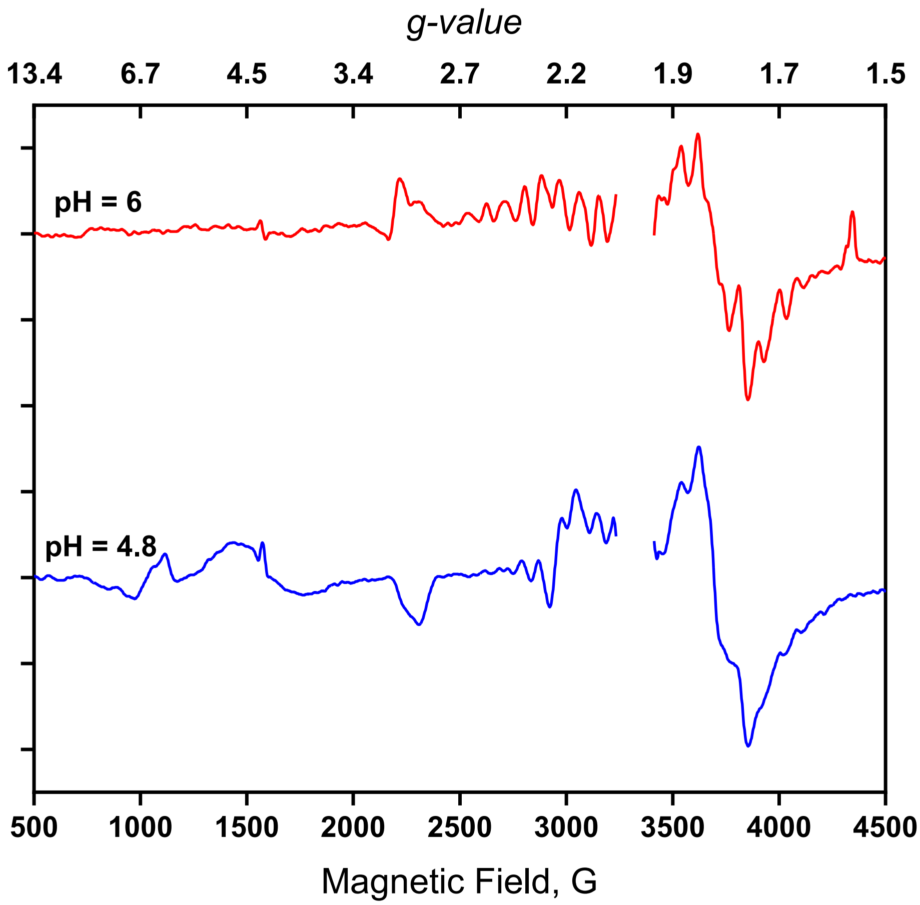

Figure S1: Acetate-induced S_2-minus-S_1 EPR spectra of PSII core complexes at different pH values. S_2-minus-S_1 state EPR spectra of 500 mM acetate-treated *Synechocystis* PSII core complexes at pH 6.0 (red) and pH 4.8 (blue).

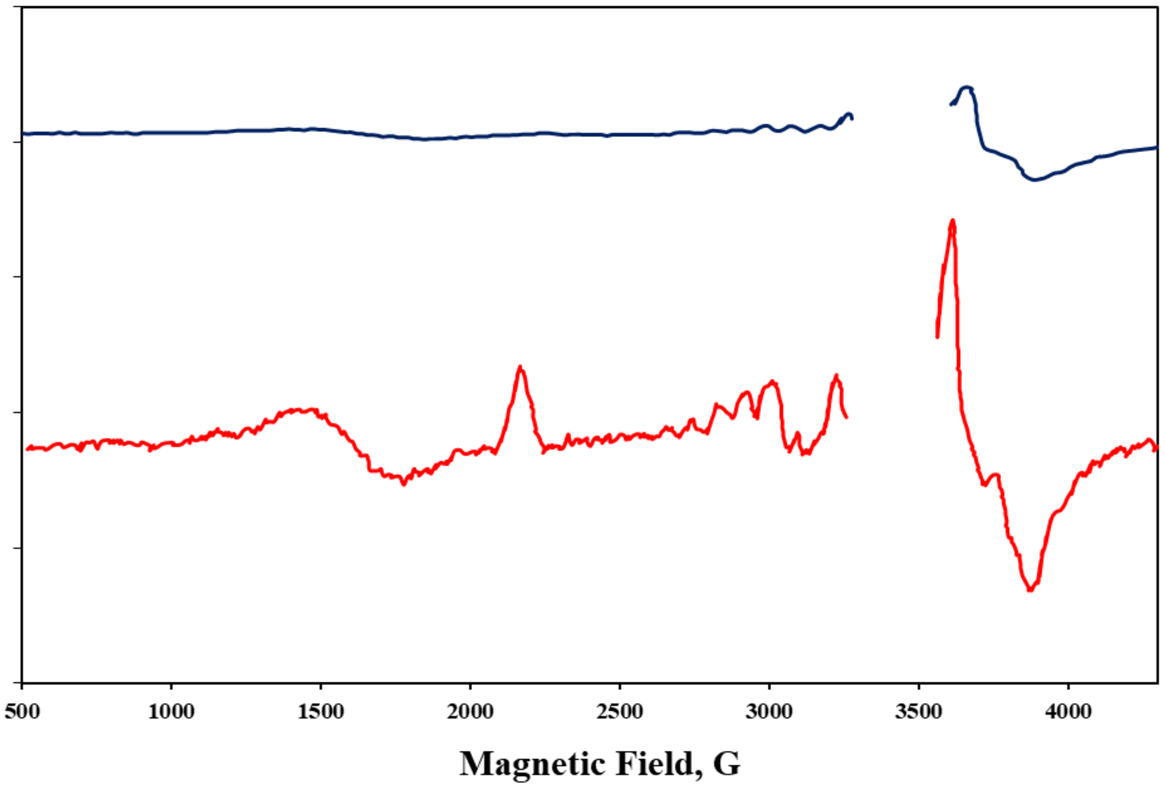

Figure S2: Comparison of acetate-treated PSII core complex and membrane S_2-minus-S_1 EPR spectra. S_2-minus-S_1 state EPR spectra of 500 mM acetate-treated *Synechocystis* PSII core complexes (blue) and 250 mM acetate-treated PSII membranes (red). Adapted from Refs.[1,2]

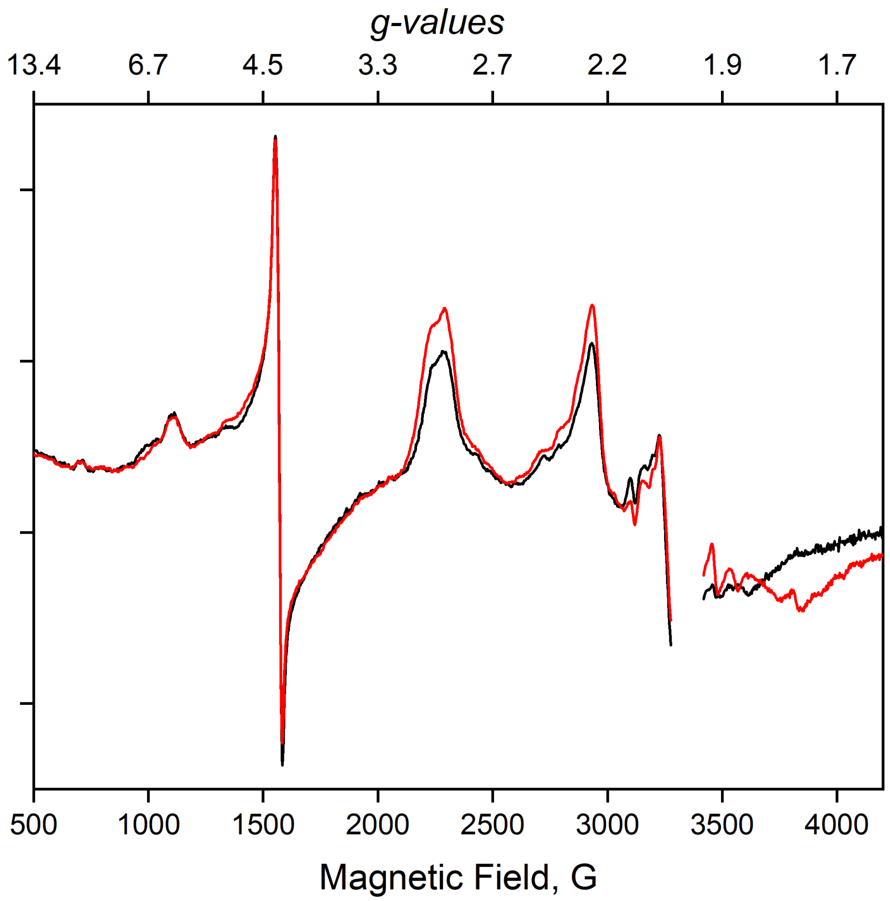

Figure S3: Acetate-treated D1-N87A PSII core complex EPR spectrum. EPR spectrum of the S_2 state in D1-N87A PSII core complexes (red) in the presence of 500 mM acetate. The dark spectrum is shown in black.

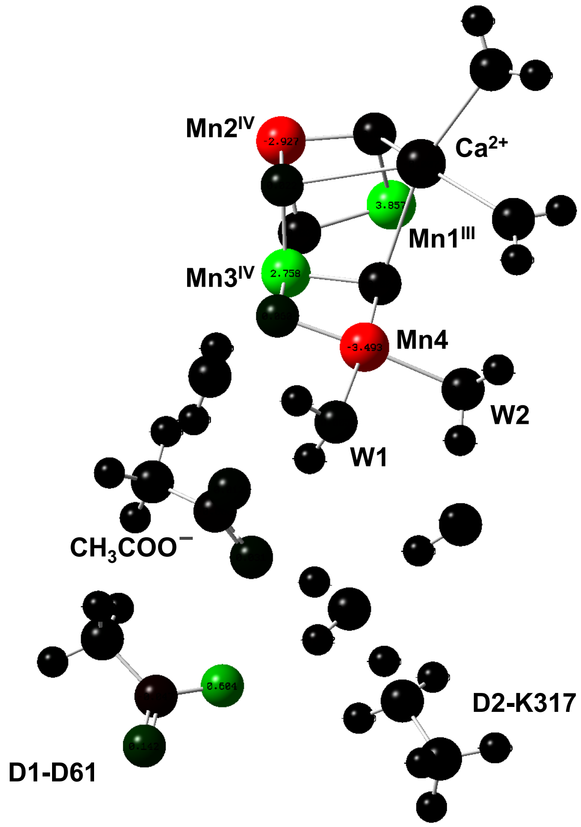

Figure S4: Spin-density redistribution upon D1-D61 deprotonation in the acetate-bound S_2 state PSII model. Mulliken spin density of the S_2 state for the $g = 2$ isomer after deletion of the D1-D61 proton, obtained from a single-point calculation. Partial radical character appears on D1-D61, with partial Mn^{III} character on Mn4, indicating that the OEC spontaneously oxidizes D1-D61 in the absence of the proton. A fully converged structure of the deprotonated D1-D61 state could not be obtained due to convergence issues, indicating instability of the state.

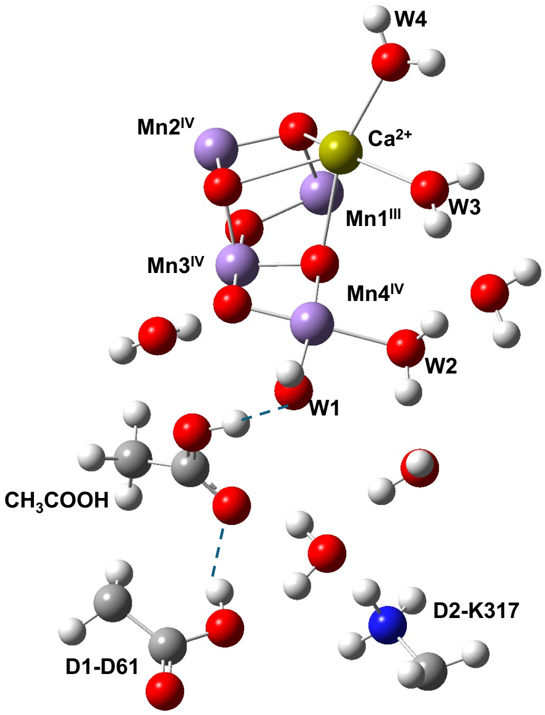

Figure S5: Alternative W1 protonation state in the acetate-bound S_2 state PSII model. QM/MM-optimized structure of acetate-bound PSII in the S_2 state for the $g = 2$ isomer with an alternative protonation state of W1. In this structure, the W1 proton is localized on acetate, resulting in a 3.6 kcal mol^{-1} increase in free energy relative to the structure with W1 as H_2O shown in Fig. 4a.

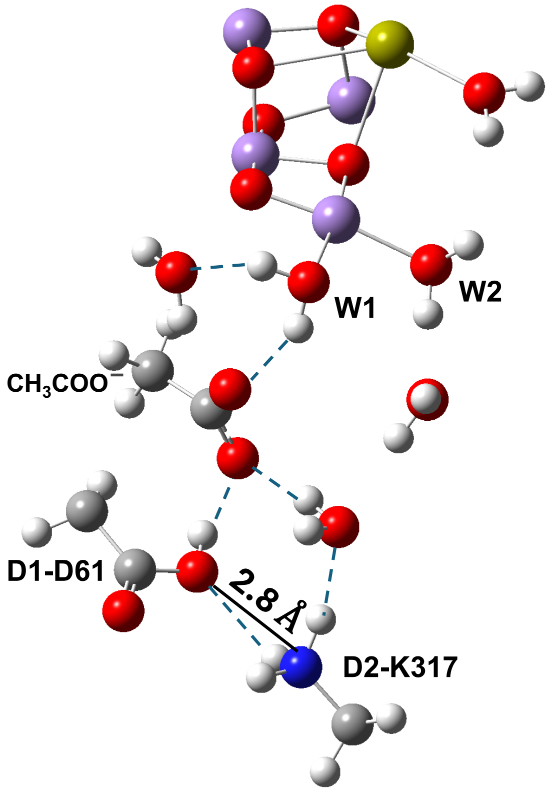

Figure S6: Alternative D2-K317 orientation in the acetate-bound PSII model. Alternative orientation of D2-K317 in the acetate-bound PSII model. The N_{K317}–O_{D61} distance is 2.8 Å, compared to 3.6 Å in the structure shown in Fig. 6a, suggesting formation of a hydrogen bond. This structure is higher in free energy by 4.9 kcal mol^{-1} and higher in electronic energy by 0.27 kcal mol^{-1}. These results suggest that this state is energetically accessible, although the energies should be interpreted with caution because these residues lie near the boundary of the QM layer.

Table S1: Summary of acetate-bound S_2 state QM/MM models.

Model	Electronic State	Protonation	Relative Energy
Low-spin acetate-bound S_2	2S + 1 = 2<br>[a]BS: $\alpha\beta\alpha\beta$<br>[b]III, IV, IV, IV<br>[c]charge = + 3	W1 = H_2O<br>acetate = CH_3COO^-<br>D1-D61 side chain = COOH	[d]ΔG = 0.0<br>ΔE = 0.0
High-spin acetate-bound S_2	2S + 1 = 6<br>BS: $\alpha\alpha\alpha\beta$<br>IV, IV, IV, III<br>charge = + 3	W1 = H_2O<br>acetate = CH_3COO^-<br>D1-D61 side chain = COOH	ΔG = 1.0<br>ΔE = 0.5
Alternative W1 protonation	2S + 1 = 2<br>BS: $\alpha\beta\alpha\beta$<br>III, IV, IV, IV<br>charge = +3	W1 = OH^-<br>acetate = CH_3COOH<br>D1-D61 side chain = COOH	ΔG = 3.6<br>ΔE = 4.2
D1-D61-deprotonated model	2S + 1 = 2<br>BS: $\alpha\beta\alpha\beta$<br>III, IV, IV, IV<br>charge = +2	W1 = H_2O<br>acetate = CH_3COO^-<br>D1-D61 side chain = COO^-	No optimized minimum obtained
D2-K317-reoriented model	2S + 1 = 2<br>BS: $\alpha\beta\alpha\beta$<br>III, IV, IV, IV<br>charge = +3	W1 = H_2O<br>acetate = CH_3COO^-<br>D1-D61 side chain = COOH	ΔG = 4.9<br>ΔE = 0.3

[a]Relative spin configurations of Mn1-4 defined by broken symmetry (BS) DFT.
[b]Formal oxidation states of Mn1-4.
[c]Charge defined for the QM region.
[d]Energies are reported relative to the optimized acetate-bound low-spin reference model in kcal mol^{-1}.

Table S2: Relevant bond distances for the hydrogen bonding network of acetate-bound QM/MM models in Å

	$S = 1/2$ S_2	$S = 5/2$ S_2	$S = 1/2$ W1=OH^-	D2-K317-reoriented model
O_{W1}–O_{WN1}	2.65	2.72	3.24	2.65
O_{W1}–$O_{CH_3COO^-}$	2.62	2.59	2.54	2.68
O_{WN1}–$O_{CH_3COO^-}$	2.73	2.75	2.78	2.76
O_{W8}–$O_{CH_3COO^-}$	2.56	2.59	2.67	2.74
$O_{D1\text{-}D61}$–$O_{CH_3COO^-}$	2.69	2.68	2.72	2.69
$O_{D1\text{-}D61}$–O_{W8}	2.90	2.82	2.82	2.91
$O_{D1\text{-}D61}$–$N_{D2\text{-}K317}$	3.67	3.63	3.67	2.86

References

(1) Boussac, A.; Sugiura, M.; Inoue, Y.; Rutherford, A. W. EPR study of the oxygen evolving complex in His-tagged photosystem II from the cyanobacterium *Synechococcus elongatus*. *Biochemistry* **2000**, *39*, 13788–13799.

(2) Kühne, H.; Szalai, V. A.; Brudvig, G. W. Competitive binding of acetate and chloride in photosystem II†. *Biochemistry* **1999**, *38*, 6604–6613.
